# *CD274* (PD-L1) negatively regulates M1 macrophage polarization in ALI/ARDS

**DOI:** 10.3389/fimmu.2024.1344805

**Published:** 2024-02-19

**Authors:** Nana Tang, Yang Yang, Yifei Xie, Guohui Yang, Qin Wang, Chang Li, Zeyi Liu, Jian-an Huang

**Affiliations:** ^1^ Department of Pulmonary and Critical Care Medicine, The First Affiliated Hospital of Soochow University, Suzhou, China; ^2^ Medical Intensive Care Unit, The Affiliated Hospital of Guizhou Medical University, Guiyang, China; ^3^ Institute of Respiratory Diseases, Soochow University, Suzhou, China

**Keywords:** severe acute respiratory distress syndrome, macrophage, M1 polarization, immune infiltration, biomarker, PD-L1, stat3, inflammation

## Abstract

**Background:**

Acute lung injury (ALI)/severe acute respiratory distress syndrome (ARDS) is a serious clinical syndrome characterized by a high mortality rate. The pathophysiological mechanisms underlying ALI/ARDS remain incompletely understood. Considering the crucial role of immune infiltration and macrophage polarization in the pathogenesis of ALI/ARDS, this study aims to identify key genes associated with both ALI/ARDS and M1 macrophage polarization, employing a combination of bioinformatics and experimental approaches. The findings could potentially reveal novel biomarkers for the diagnosis and management of ALI/ARDS.

**Methods:**

Gene expression profiles relevant to ALI were retrieved from the GEO database to identify co-upregulated differentially expressed genes (DEGs). GO and KEGG analyses facilitated functional annotation and pathway elucidation. PPI networks were constructed to identify hub genes, and differences in immune cell infiltration were subsequently examined. The expression of hub genes in M1 versus M2 macrophages was evaluated using macrophage polarization datasets. The diagnostic utility of *CD274* (PD-L1) for ARDS was assessed by receiver operating characteristic (ROC) analysis in a validation dataset. Experimental confirmation was conducted using two LPS-induced M1 macrophage models and an ALI mouse model. The role of *CD274* (PD-L1) in M1 macrophage polarization and associated proinflammatory cytokine production was further investigated by siRNA-mediated silencing.

**Results:**

A total of 99 co-upregulated DEGs were identified in two ALI-linked datasets. Enrichment analysis revealed that these DEGs were mainly involved in immune-inflammatory pathways. The following top 10 hub genes were identified from the PPI network: *IL-6*, *IL-1β*, *CXCL10*, *CD274*, *CCL2*, *TLR2*, *CXCL1*, *CCL3*, *IFIT1*, and *IFIT3*. Immune infiltration analysis revealed a significantly increased abundance of M1 and M2 macrophages in lung tissue from the ALI group compared to the control group. Subsequent analysis confirmed that *CD274* (PD-L1), a key immunological checkpoint molecule, was highly expressed within M1 macrophages. ROC analysis validated *CD274* (PD-L1) as a promising biomarker for the diagnosis of ARDS. Both *in vitro* and *in vivo* experiments supported the bioinformatics analysis and confirmed that the JAK-STAT3 pathway promotes *CD274* (PD-L1) expression on M1 macrophages. Importantly, knockdown of *CD274* (PD-L1) expression potentiated M1 macrophage polarization and enhanced proinflammatory cytokines production.

**Conclusion:**

This study demonstrates a significant correlation between *CD274* (PD-L1) and M1 macrophages in ALI/ARDS. *CD274* (PD-L1) functions as a negative regulator of M1 polarization and the secretion of proinflammatory cytokines in macrophages. These findings suggest potential new targets for the diagnosis and treatment of ALI/ARDS.

## 1 Introduction

Acute lung injury (ALI)/acute respiratory distress syndrome (ARDS) is a common clinical syndrome, induced by various pulmonary and extrapulmonary factors. This syndrome is characterized by rapid onset and is associated with a high mortality rate, estimated at around 40% (1, 2). Although the precise pathophysiology of ALI/ARDS has not been fully elucidated, it is evident that immune cell infiltration plays a pivotal role in its progression (3–6).

Macrophages, a major cell type in innate immunity, are abundantly distributed within the lung microenvironment and exhibit extensive plasticity and can adopt different phenotypes in response to various stimuli (7). Extensive evidence indicates that macrophage polarization plays a critical role in the onset and progression of ALI/ARDS (8–10). Programmed death-ligand 1 (PD-L1), encoded by the *CD274* gene, is found in various cellular environments (11, 12). Acting as an immune-checkpoint molecule, *CD274* (PD-L1) is instrumental in attenuating the immune response, thereby impacting not only the pathogenesis of tumors but also influencing the course of non-malignant conditions (13–16). However, the specific function of *CD274* (PD-L1) in the pathophysiology of ALI/ARDS remains a complex puzzle. Administering soluble PD-L1 to ALI mice has been shown to attenuate inflammatory lung damage and improve overall survival (17). In contrast, Wang and colleagues found that elevated *CD274* (PD-L1) expression in human neutrophils impedes cellular apoptosis, eliciting PI3K-dependent AKT phosphorylation, and worsening lung injury and raising mortality rates (18). Zhu et al. demonstrated that *CD274* (PD-L1) amplifies lung injury by persistently releasing neutrophil extracellular traps (NETs) and manipulating autophagy (19). These conflicting findings highlight the need for further research to fully understand the role of *CD274* (PD-L1) in ALI/ARDS. Additionally, the relationship between *CD274* (PD-L1) and macrophage polarization in ALI/ARDS and the potential diagnostic utility of *CD274* (PD-L1) in ALI/ARDS remains less investigated.

**Figure 1:**
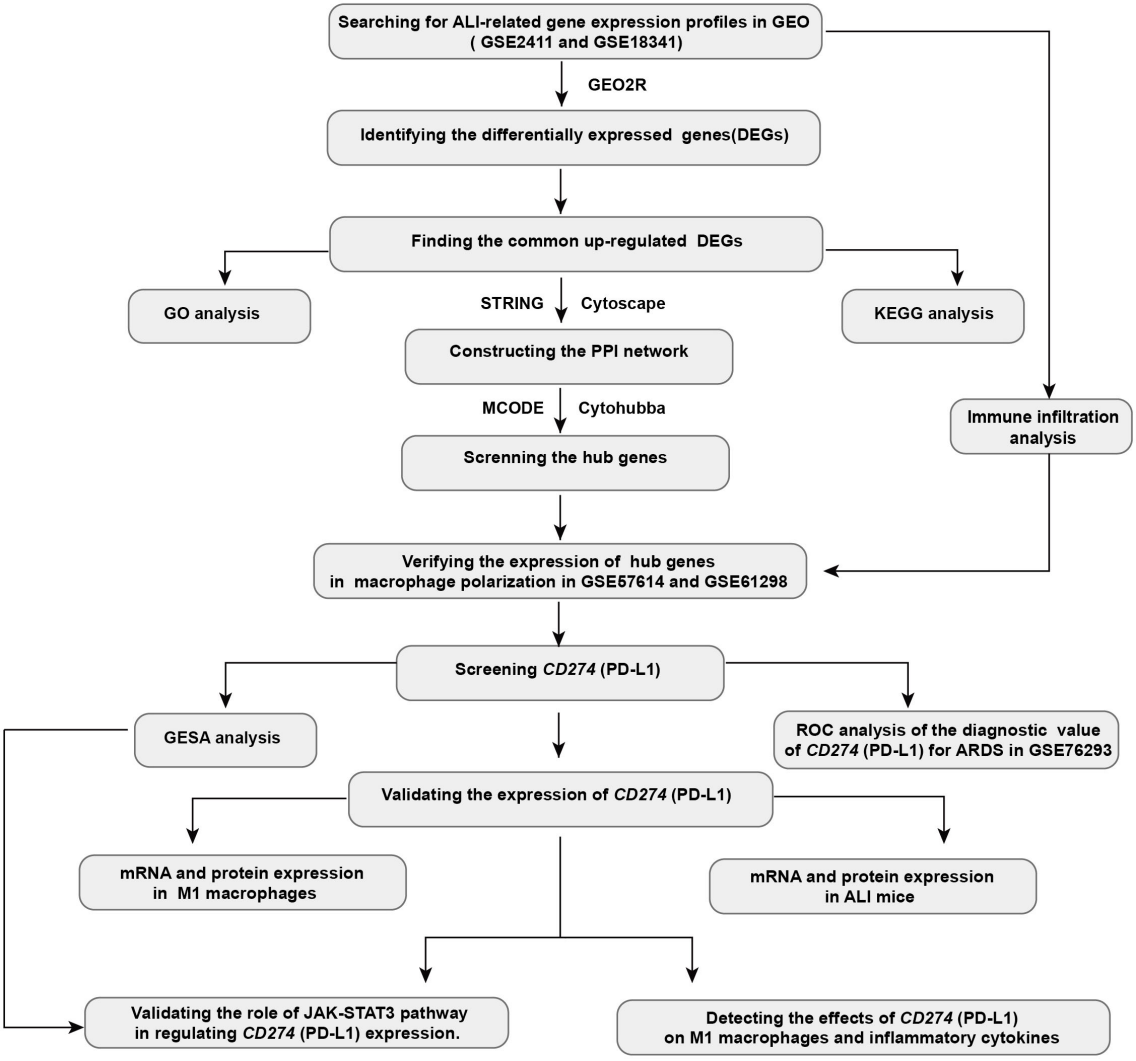
Workflow chart for this study.

In this study, we employed bioinformatics approach to analyze relevant datasets on ALI/ARDS and macrophage polarization. The analysis revealed that *CD274* (PD-L1) expression is elevated and serves as a pivotal molecule correlating with M1 macrophage and ALI/ARDS. The results of the bioinformatics analyses were further validated through correlation experiments in M1 macrophage models and mouse models of the early phase of ALI. Further investigations have revealed that the JAK-STAT3 pathway upregulates *CD274* (PD-L1) expression. In addition, *CD274* (PD-L1) negatively regulates M1 macrophage polarization and proinflammatory cytokine production. Collectively, these findings suggest a negative role of *CD274* (PD-L1) on M1 macrophage function. *CD274* (PD-L1) may have potential value in the diagnosis and treatment of ALI/ARDS. The workflow chart for this study is displayed in **Figure 1**.

## 2 Materials and methods

### 2.1 Dataset collection and DEG identification

Gene expression profiles of lipopolysaccharide (LPS)-induced ALI in C57BL/6 mice (GSE2411 and GSE18341), human macrophage polarization (GSE57614 and GSE61298), and ARDS patients (GSE76293) were obtained from the Gene Expression Omnibus (GEO) database. GSE2411 and GSE18341 were utilized to identify differentially expressed genes (DEGs) and to perform immune infiltration analyses. GSE57614 and GSE61298 were used to validate M1 macrophage polarization. GSE76293 served as the validation cohort to assess the diagnostic significance of *CD274*. **Supplementary Table 1** provides information on these datasets. The DEGs were identified and analyzed via GEO2R. A significance threshold of |log2(fold change)| ≥1.5 and an adjusted *p*-value < 0.05 was applied to determine statistical significance. Volcano plots of DEGs and Venn diagrams of co-upregulated DEGs were displayed using the ‘ggplot2’ package in the R software environment.

### 2.2 Functional enrichment analysis of co-upregulated DEGs

The identified co-upregulated DEGs were analyzed using the Gene Ontology (GO) and Kyoto Encyclopedia of Genes and Genomes (KEGG) databases to establish a complete functional annotation. GO analysis encompassed three domains: Biological Process (BP), Cellular Component (CC), and Molecular Function (MF). Functional enrichment analysis was performed using the Database for Annotation, Visualization, and Integrated Discovery web tool. The outcome of the analysis was visualized using the ‘GOplot’ and ‘ggplot2’ packages within the R programming environment.

### 2.3 Construction of PPI networks and identification of hub genes

The protein-protein interaction (PPI) network of the co-upregulated DEGs was constructed using the STRING website. The PPI network outcomes, maintaining at least a 0.4 minimum interaction score, were then visualized via Cytoscape version 3.9.1. Hub genes screening was facilitated by MCODE and CytoHubba plug-ins.

### 2.4 Analysis of immune infiltration

The ImmuCellAI-mouse, available at http://bioinfo.life.hust.edu.cn/ImmuCellAI-mouse, is a useful resource for determining the abundance of various immune (sub-)cell types from gene expression data through a three-tier hierarchical approach. Immune cell type reference profiles and sturdy marker gene sets were curated. The abundance of cells in each layer was predicted individually by computing the ssGSEA enrichment score of the expression deviation profile per cell type (20). Expression matrices were uploaded to the ImmuCellAI-mouse online server for immune infiltration analysis.

### 2.5 Verifying expression of hub genes in macrophage polarization

As the validation cohort, two datasets related to human macrophage polarization, GSE57614 and GSE61298, were used to identify differences in hub genes expression between M1 and M2 macrophages. This approach helped distinguish the hub genes relevant to M1 polarization in ALI. The heatmap, plotted using the ‘ComplexHeatmap’ packages in the R programming environment, visually reflects the level of differential expression of these genes.

### 2.6 Validation of the diagnostic value of *CD274* in ARDS

The diagnostic potential of *CD274* in relation to ARDS was investigated using the GSE76293 dataset. A significance threshold of *p* < 0.05 was applied to evaluate the diagnostic performance of *CD274*. Receiver operating characteristic (ROC) curves were generated and analyzed to further assess the diagnostic capability. Finally, the area under the curve (AUC) value, ranging from 0 to 1, was calculated after analysis.

### 2.7 Gene set enrichment analysis

To elucidate the association between *CD274* and signaling pathways, the expression datasets of ALI were grouped according to the median value of *CD274* expression. Enrichment analysis was conducted on different subgroups using GSEA version 4.3.1 software, with an adjusted *p*-value < 0.05 as the threshold for significance (21).

### 2.8 Cell culture, treatment, and siRNA transfection

The RAW264.7 murine macrophage cell line was acquired from Procell Co. Ltd (Wuhan, China) and cultured in Dulbecco’s Modified Eagle Medium supplemented with 10% fetal bovine serum (FBS) and 1% penicillin-streptomycin, under a humidified atmosphere containing 5% CO_2_ at 37°C. Bone marrow was extracted from the femurs and tibias of C57BL/6 mice. The extracted bone marrow cells were filtered through a 70-μm cell strainer and centrifuged. After lysing the red blood cells, the resulting cells were cultured in RPMI 1640 medium supplemented with 10% FBS, 1% penicillin-streptomycin, and 20 ng/mL of recombinant murine macrophage colony-stimulating factor (M-CSF, PeproTech, USA). The medium was refreshed on day 3 and day 6 to facilitate macrophage differentiation. On day 7, non-adherent cells were removed, leaving surface-adherent bone marrow-derived macrophages (BMDMs). BMDMs were identified using flow cytometry by detecting F4/80 (**Supplementary Figure 1**).

The cells were treated with LPS (500 ng/mL) derived from *E. coli* 055:B5 (Sigma-Aldrich, USA) for 12 hours to induce the M1 phenotype. The cells in the inhibitor group were pretreated with Stattic (MCE, China), a potent inhibitor of STAT3 phosphorylation, for 1 hour (22, 23). BMDMs were transfected with *CD274*-siRNA (si*CD274*-1 and si*CD274*-2) or negative control siRNA (siNC) using HiPerFect Transfection Reagent (QIAGEN, Germany), following the manufacturer’s instructions. The siRNA target sequences are listed in **Supplementary Table 2**.

### 2.9 ALI mouse models

The Soochow University Animal Ethics Committee-approved Guide for the Care and Use of Laboratory Animals was followed during the animal procedures. Male C57BL/6 mice weighing 20–22 g and aged six to eight weeks were housed in Soochow University’s Experimental Animal Center. Mice were randomly assigned to either the control group or the ALI group (n=6). Early phase ALI was induced in the ALI group mice through intraperitoneal administration of 10 mg/kg of LPS for 12 hours. Meanwhile, the mice in the control group received an equivalent amount of saline.

### 2.10 Histology and immunohistochemistry

Mouse lung tissues were fixed in 4% paraformaldehyde, dehydrated through a graded series of alcohols, embedded in paraffin, and sectioned at 5 µm. The sections were then stained with hematoxylin and eosin (H&E) for histological examination. For immunohistochemistry, the sections were deparaffinized, rehydrated, washed, and subjected to antigen retrieval. The sections were subsequently incubated with 5% bovine serum albumin (BSA) to block non-specific binding and then with a PD-L1 antibody at 4°C overnight. Immunoreactivity was detected using a horseradish peroxidase (HRP)-conjugated secondary antibody followed by visualization with 3,3’-diaminobenzidine tetrahydrochloride (DAB)(ZSGB-BIO, China).

### 2.11 qRT-PCR analysis

RNA from cells, lung tissue homogenates, and peripheral blood samples of mice were extracted utilizing TRIzol reagent (Vazyme, China) according to the manufacturer’s protocol. Extracted RNA was then reverse-transcribed into complementary DNA (cDNA) using reverse transcription reagents (Vazyme, China). Quantitative real-time PCR (qRT-PCR) analysis was carried out using SYBR Premix ExTaq™ (Vazyme, China). **Supplementary Table 3** lists the primer sequences used for each target gene. Relative mRNA expression levels were normalized to *GAPDH* mRNA and the 2^-ΔΔ^CT method was employed to calculate the fold change in gene expression.

### 2.12 Western blotting analysis

Proteins from lung tissues and cells were extracted using RIPA buffer (Beyotime, China). The total protein concentration in the lysates was measured using the BCA protein assay kit (Beyotime, China). The equivalent protein was loaded onto a 10% SDS-PAGE gel and then electrophoretically transferred to PVDF membranes. Subsequently, the membranes were blocked with 5% BSA for 1 hour at room temperature, followed by overnight incubation at 4°C with specific primary antibodies. After washing with TBST buffer, the membranes were incubated with an HRP-conjugated IgG antibody for two hours at room temperature. Finally, the antigen was detected using an imaging system Tanon 5260 (Tanon, China) and quantitatively analyzed using ImageJ software (Bethesda, USA). All the experiments were repeated a minimum of three times.

### 2.13 Flow Cytometry

The cultured cells were collected and incubated with anti-FcγR blocking mAb (clone 2.4G2) at 4°C for 30 minutes. The cells were then stained with APC-anti-F4/80 and PE-anti-CD86 at 4°C for 30 minutes. The stained cells were washed and analyzed using the FACS Canto II flow cytometer (BD Biosciences, USA) with FlowJo seven software (Tree Star, USA). The antibodies utilized for Western blotting, immunohistochemistry, and flow cytometry in this study are listed in **Supplementary Table 4**.

### 2.14 Enzyme-linked immunosorbent assay

The concentrations of the inflammatory cytokines TNF-α and IL-6 were quantified using ELISA, following the manufacturer’s instructions (BOSTER, China) after collecting the cell supernatants.

### 2.15 Statistical analyses

All data were analyzed using the standard two-tailed unpaired Student’s t-test between two groups and a one-way ANOVA followed by Tukey’s multiple comparisons test for comparison of three or more groups. Statistical analysis was performed using GraphPad Prism (version 8.0) and statistically analyzed data are presented as mean ± SEM. *p*-values < 0.05 were considered to indicate statistically significant results.

## 3 Results

### 3.1 Identification of DEGs between ALI and healthy controls

**Figure 2:**
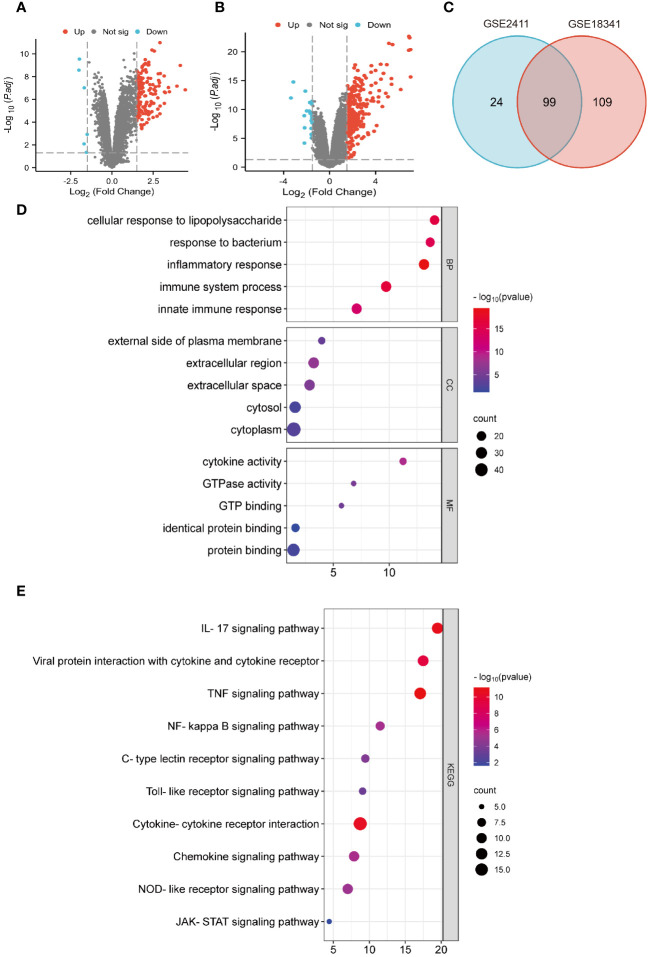
Identification of DEGs and functional enrichment analyses. **(A)** Volcano plot of GSE 2411. **(B)** Volcano plot of GSE 18341. **(C)** Venn diagrams of co-upregulated DEGs. **(D)** Bubble plots of GO analysis. **(E)** Bubble plots of KEGG analysis.

To identify potential genes associated with ALI, two GEO datasets (GSE2411 and GSE18341) were analyzed. The result revealed a total of 128 DEGs (123 upregulated and five downregulated) in the GSE2411 dataset (**Figure 2A**) and 227 DEGs (208 upregulated and 19 downregulated) in the GSE18341 dataset (**Figure 2B**). A total of 99 co-upregulated DEGs were identified between the two datasets (**Figure 2C**).

### 3.2 GO and KEGG pathway analyses of co-upregulated DEGs

Functional enrichment analyses of co-upregulated DEGs were performed using GO and KEGG pathway analyses. In GO analysis, the BP category was predominantly enriched in processes such as response to LPS, inflammatory response, immune system process, and innate immune response. In the CC category, the main enrichments were observed in the cytoplasm and extracellular regions. The MF category exhibited enrichment in processes such as protein binding, cytokine activity, and GTP binding (**Figure 2D**). Additionally, KEGG pathway analysis revealed significant enrichments in pathways such as the IL-17 signaling pathway, viral protein interaction with cytokines and cytokine receptors, the TNF signaling pathway, NF-κB signaling pathway, and cytokine-cytokine receptor interaction (**Figure 2E**).

### 3.3 PPI network construction and identification of hub genes

**Figure 3:**
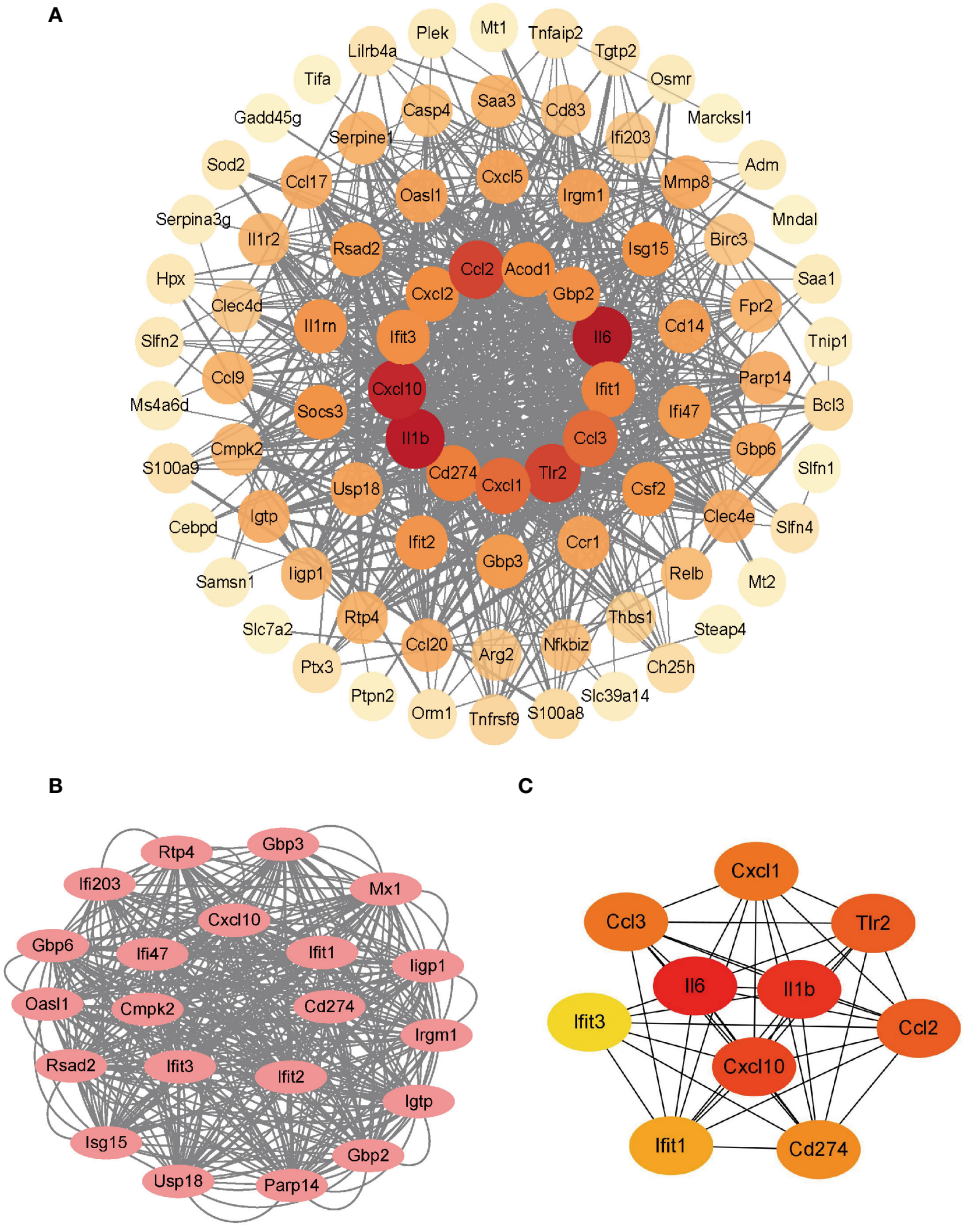
Construction of PPI network and identification of hub genes. **(A)** PPI network of co-upregulated DEGs. **(B)** Genes in the module with the highest MCODE value. **(C)** Top 10 hub genes.

The PPI network of the co-upregulated DEGs was constructed using the STRING database (**Figure 3A**). By utilizing the Cytoscape plugin MCODE, the most significant clustering module was extracted from the entire network, consisting of 21 nodes (score:20.1) (**Figure 3B**). Subsequently, the CytoHubba plugin, which identifies essential nodes in the network, was used to identify the top 10 hub genes: *IL-6*, *IL-1β*, *CXCL10*, *CD274*, *CCL2*, *TLR2*, *CXCL1*, *CCL3*, *IFIT1*, and *IFIT3* (**Figure 3C**).

### 3.4 Immune cell Infiltration analysis

**Figure 4:**
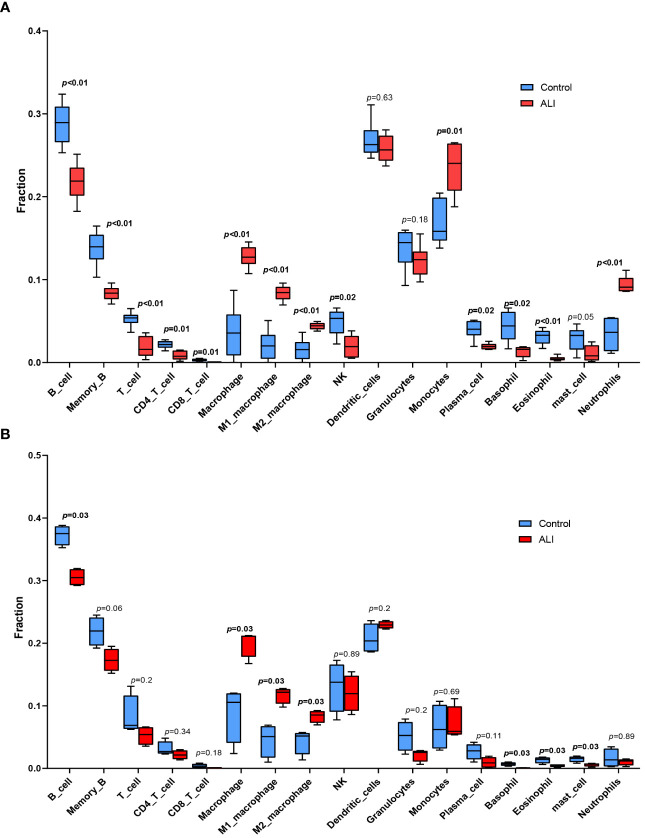
Immune cell infiltration analysis. **(A)** Immune cell infiltration analysis in GSE2411. **(B)** Immune cell infiltration analysis in GSE18341.

The co-upregulated DEGs, as identified by gene function enrichment analyses, were primarily associated with immune and inflammation-related signaling pathways. The ImmuCellAI-mouse algorithm was utilized to evaluate the distribution of immune cells in the GSE2411 and GSE18341 datasets, enabling further investigation of differences in immune cell infiltration between the ALI and control groups. Significant differences were observed in the infiltration of four immune cell types in the lung tissues of both groups (*p* < 0.05). Specifically, there was increased prevalence of macrophages within the ALI group relative to controls, with a higher relative abundance of M1 macrophages than M2 macrophages. Conversely, the control group was characterized by a greater proportion of B cells, basophils, and eosinophils (**Figures 4A, B**).

### 3.5 Detecting expression of hub genes in macrophage-related datasets

**Figure 5:**
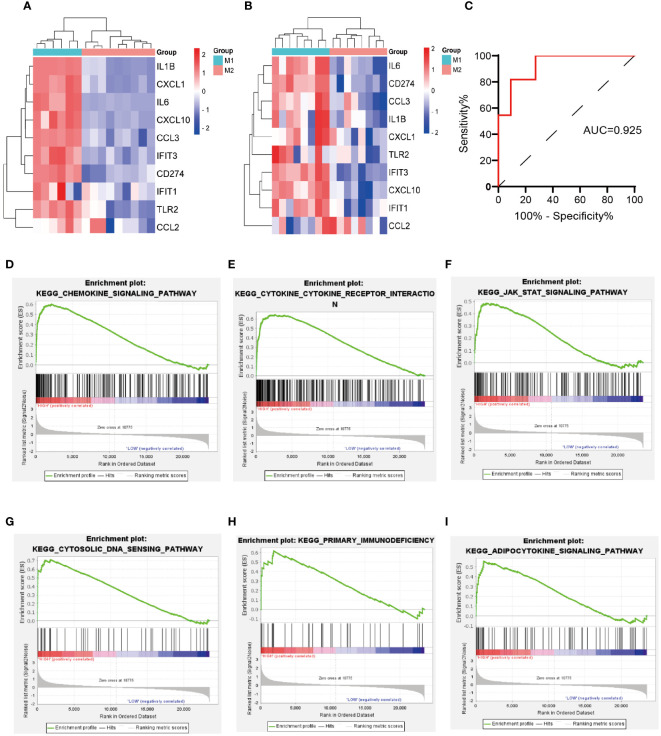
Validation of hub genes expression in macrophage polarization-related datasets, validation of the diagnostic value of *CD274* for ARDS, and GSEA of *CD274*. **(A)** Validation of hub genes expression in GSE61298. **(B)** Validation of hub genes expression in GSE57614. **(C)** Evaluation of the diagnostic value of *CD274* for ARDS using ROC curves in GSE76293. **(D–I)** GSEA of *CD274*.

Two macrophage-related datasets, GSE61298 and GSE57614, were used to validate differences in the expression of the identified hub genes across varying macrophage polarization phenotypes. In both datasets, multiple hub genes, particularly *IL-6*, *CD274*, *IFIT3*, and *CXCL10*, exhibited significantly higher expression in M1 macrophages than in M2 macrophages (**Figures 5A, B**). *IL-6* and *CXCL10* have been confirmed to be signature genes of M1 macrophages (24–26). We are concurrently investigating the role of IFIT3 in the pathogenesis of ALI/ARDS in a separate study. Scant research has delved into the correlation between *CD274* and M1 macrophages in ALI/ARDS. Thus, *CD274* was selected for further investigation and validation in this study.

### 3.6 Evaluating the diagnostic performance of *CD274* in ARDS

The diagnostic accuracy of *CD274* for ARDS was assessed by ROC curve analysis using an external validation cohort, GSE76293. The AUC was found to be 0.925, with a notable 95% confidence interval ranging from 0.818 to 1.000 (**Figure 5C**), demonstrating the substantial diagnostic potential of *CD274* in ARDS.

### 3.7 GSEA of *CD274*

Through GSEA analysis of *CD274*-associated signaling pathways (**Figures 5D–I**), it was revealed that the primary enriched pathways were the chemokine signaling pathway, cytokine-cytokine receptor interaction, JAK-STAT signaling pathway, cytosolic-DNA sensing pathway, primary immunodeficiency, and the adipocytokine signaling pathway.

### 3.8 Assessing *CD274*(PD-L1*)* expression in LPS-induced M1 macrophage models

**Figure 6:**
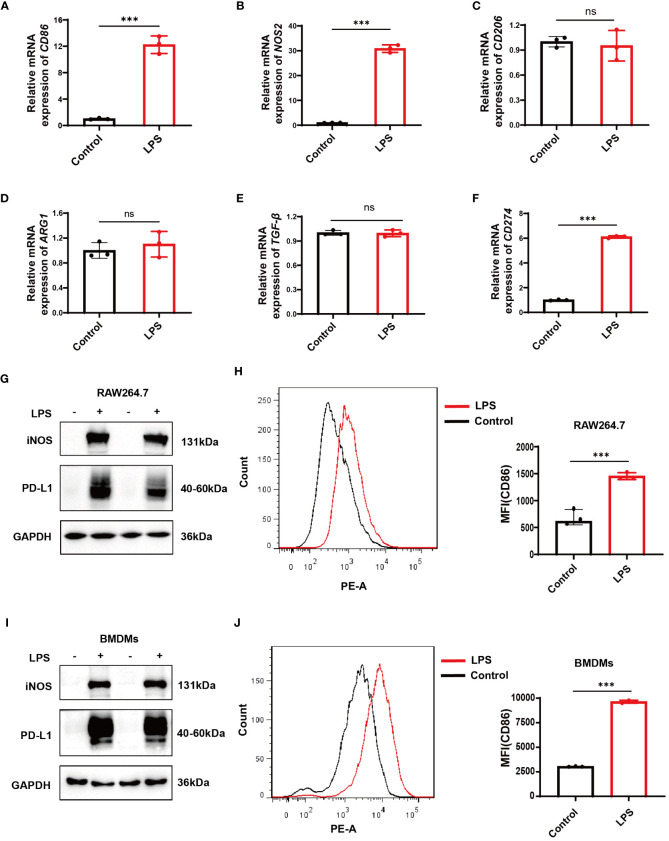
Verification of *CD274* (PD-L1) expression in M1 macrophage models through LPS stimulation (500 ng/ml) for 12 hours. **(A, B)** qPCR analysis of M1 macrophage markers (*CD86* and *NOS2*) in RAW264.7 cells, under control and LPS conditions. **(C–E)** qPCR analysis of M2 markers (*CD206*, *ARG1*, and *TGF*-*β*) in RAW264.7 cells under control and LPS conditions. **(F)** qPCR analysis of *CD274* mRNA levels in RAW264.7 cells under control and LPS conditions. **(G)** Western blotting analysis of PD-L1 and iNOS protein expression in RAW264.7 cells under control and LPS conditions. **(H)** Flow cytometric analysis of CD86 mean fluorescence intensity (MFI) on RAW264.7 cells under control and LPS conditions. **(I)** Western blotting of PD-L1 and iNOS protein levels in BMDMs, under control and LPS conditions. **(J)** Flow cytometry displaying CD86 MFI on F4/80^+^ BMDMs, under control and LPS conditions. (ns, not significant, ***p < 0.001).

After 12 hours of stimulation with LPS at a dose of 500ng/mL in RAW264.7 cells and BMDMs, qPCR results in RAW264.7 cells revealed a significant increase in the mRNA expression levels of *CD86* and inducible nitric oxide synthase (*NOS2*, iNOS), the markers of M1 macrophages (**Figures 6A, B**). At the same time, no significant change was observed in the expression levels of the M2 markers *CD206*, arginase 1 (*ARG1*), and transforming growth factor beta (*TGF*-*β*) (**Figures 6C–E**). Both RAW264.7 cells and BMDMs showed a significant increase in iNOS and CD86 protein levels as demonstrated by Western blotting and flow cytometry, respectively (**Figures 6G–J**). These data suggest a successful establishment of LPS-induced M1 polarization models in RAW264.7 cells and BMDMs. Correspondingly, a significant increase in *CD274* (PD-L1) mRNA and protein levels was observed along with M1 polarization in both cell types (**Figures 6F, G, I**).

### 3.9 Assessing *CD274* (PD-L1*)* expression in LPS-induced early phase ALI mouse model

**Figure 7:**
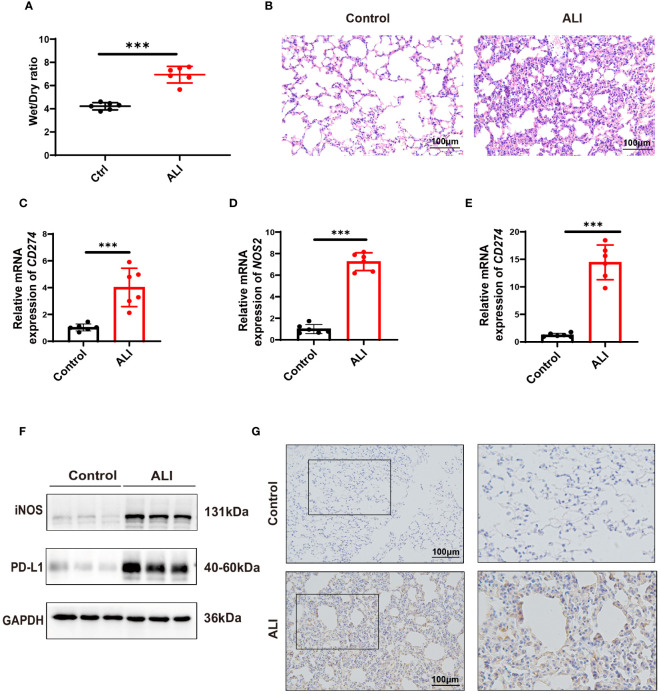
Verification of *CD274*(PD-L1) expression in ALI mice by intraperitoneal LPS (10 mg/kg) for 12 hours. **(A)** The degree of pulmonary edema was assessed in both the control and ALI groups using the lung Wet/Dry ratio. **(B)** Pathological changes in lung tissue were detected in both the control and ALI groups using H&E staining. scale bar =100 μm. **(C, D)** qPCR analysis of *CD274* and *NOS2* mRNA expression in lung tissues from control and ALI groups. **(E)** qPCR analysis of *CD274* mRNA expression in peripheral blood from control and ALI groups. **(F)** Western blotting analysis of PD-L1 and iNOS protein expression in lung tissues from control and ALI groups. **(G)** The expression of PD-L1 in lung tissues from control and ALI groups were analyzed by IHC. scale bar =100 μm. (***p < 0.001).

The severity of lung injury was quantified by lung wet/dry weight ratio and H&E staining. The results showed that the lung tissues of LPS-challenged mice exhibited significant edema, hyperemia, inflammatory cell infiltration, and alveolar septal thickening compared with the control group. These observations confirmed the successful establishment of an early phase ALI model (**Figures 7A, B**). In the ALI group, the expressions of both *CD274* (PD-L1) and *NOS2* (iNOS) were significantly increased in lung tissues at the mRNA and protein levels (**Figures 7C, D, F, G**), consistent with previous bioinformatics findings. In addition, qPCR analysis revealed higher levels of *CD274* in the peripheral blood of ALI mice compared to the control group (**Figure 7E**), suggesting the potential of *CD274* (PD-L1) as an early diagnostic biomarker for ALI/ARDS.

### 3.10 JAK-STAT3 pathway upregulates PD-L1 expression in LPS-induced M1 macrophages

**Figure 8:**
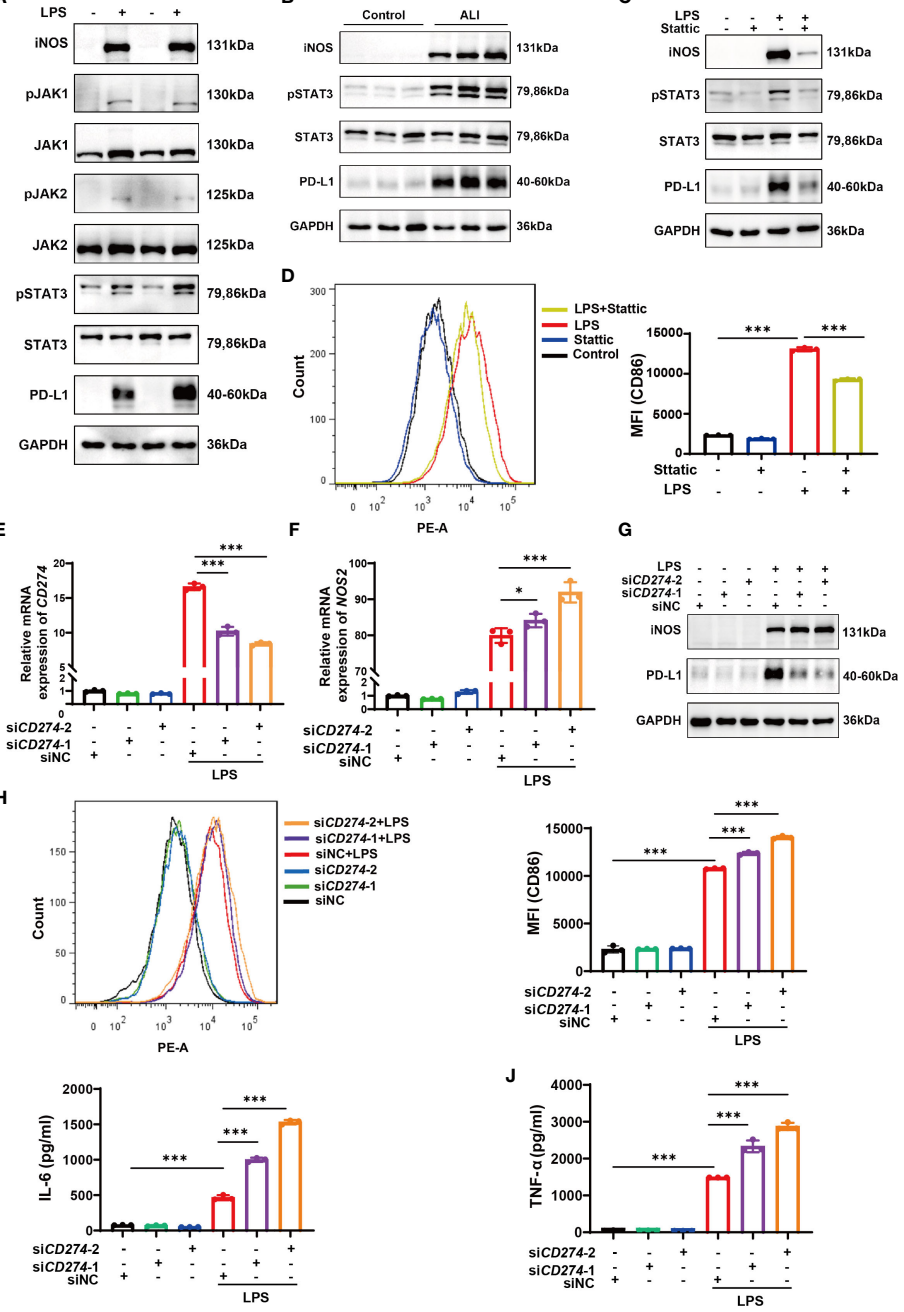
The JAK-STAT3 pathway upregulates *CD274* (PD-L1) expression in LPS-induced M1 macrophages. *CD274* (PD-L1) negatively regulates M1 polarization and the production of proinflammatory cytokines in BMDMs induced by LPS. **(A)** Protein expressions of JAK1,2-STAT3 signaling molecules were determined in BMDMs from both the control and LPS groups by Western blotting. **(B)** Protein expressions of pSTAT3 and STAT3 were determined in lung tissues from control and ALI groups by Western blotting. **(C)** Protein expressions of PD-L1, pSTAT3, STAT3, and iNOS were analyzed in BMDMs pretreated with Stattic (2.0 μM) for 1 hour and then stimulated with LPS (500 ng/mL) for 12 hours by Western blotting. **(D)** Flow cytometry displaying CD86 MFI on F4/80^+^ BMDMs that were pretreated with Stattic and then stimulated with LPS. **(E, F)** mRNA expression of *CD274* and *NOS2* was analyzed by qPCR in BMDMs that were transfected with *CD274* siRNA (si*CD274*-1 and si*CD274*-2) or negative control siRNA (siNC), followed by LPS stimulation for 12 hours. **(G)** Protein levels of PD-L1 and iNOS were analyzed by Western blotting in BMDMs transfected with *CD274* siRNA or siNC, followed by LPS stimulation. **(H)** Flow cytometry displaying CD86 MFI on F4/80^+^ BMDMs, transfected with *CD274* siRNA or siNC, followed by LPS stimulation. **(I, J)** The levels of IL-6 and TNF-α were analyzed by ELISA in the supernatant of BMDMs transfected with *CD274* siRNA or siNC, followed by LPS stimulation (*p < 0.05, ***p < 0.001).

Enrichment analysis conducted via GSEA hinted at a correlation between elevated *CD274* expression and the JAK-STAT signaling pathway. Since STAT3 has been shown to regulate PD-L1 expression in tumor cells (27–29), it is important to investigate whether this molecule also regulates macrophage PD-L1 expression in ALI/ARDS. Therefore, we selected the JAK-STAT3 signaling pathway for further investigation. Western blotting analysis demonstrated a conspicuous increase in the phosphorylation levels of JAK1, JAK2, and STAT3 in LPS-induced M1 polarized BMDMs (**Figure 8A**). Similarly, lung tissue samples from mice with ALI showed marked elevation in phosphorylated STAT3 (**Figure 8B**). To delineate the role of STAT3 phosphorylation, BMDMs were pretreated with Stattic, an inhibitor of STAT3 phosphorylation at Y705 and S727, followed by LPS stimulation. Subsequent Western blotting and flow cytometry assays revealed a notable downregulation of iNOS and CD86 expression in BMDMs, coupled with diminished PD-L1 protein levels (**Figures 8C, D**). Taken together, the data indicates that LPS stimulation results in the activation of the JAK-STAT3 signaling pathway, which in turn promotes M1 polarization in macrophages and enhances PD-L1 expression.

### 3.11 *CD274*(PD-L1) negatively regulates LPS-Induced M1 Macrophage Polarization and proinflammatory cytokine production

To investigate the effect of *CD274* (PD-L1) on macrophage M1 polarization and subsequent inflammatory responses, we transfected BMDMs with siRNA duplexes designed to knock down *CD274* expression. As shown in **Figures 8E, G**, treatment with both si*CD274*-1 and si*CD274*-2 successfully knocked down *CD274* (PD-L1) expression at both mRNA and protein levels. Notably, the effection of gene silencing was significantly enhanced upon LPS stimulation. The qPCR, Western blotting, and flow cytometry assays showed that silencing *CD274* (PD-L1) did not affect the basal expression of *NOS2* (iNOS) and CD86 in BMDMs. However, upon LPS-induced M1 polarization of BMDMs, increased expression levels of both M1 phenotypic markers were observed (**Figures 8F–H**). Additionally, the secretion of inflammatory cytokines in BMDMs was assessed by ELISA. The data show that knocking down *CD274* (PD-L1) significantly increased the expression of IL-6 and TNF-α in M1 macrophages following LPS stimulation (**Figures 8I, J**). These results suggest that *CD274* (PD-L1) has a negative regulatory effect on LPS-induced M1 polarization and the consequent production of pro-inflammatory cytokines by macrophages.

## 4 Discussion

ALI/ARDS presents profound challenges in intensive care medicine due to its high morbidity and mortality (1, 2). Despite ongoing research, the pathogenesis of ALI/ARDS is yet to be fully elucidated. A consensus holds that excessive inflammation is central to its pathophysiology, involving several effector and target cells (3, 30). Within the pulmonary milieu, macrophages—vital to the innate immune defense—play a multifaceted role in the development of ALI/ARDS (31–33). Alveolar macrophages (AMs) and interstitial macrophages (IMs) are abundant in the lungs and are pivotal to this process (34). These macrophages can be characterized by two principal polarization states: M1 and M2. M1 macrophages are pro-inflammatory and play essential roles in the defense against infection, while M2 macrophages are involved in anti-inflammatory activities and facilitate tissue repair (7, 35–37). An imbalance between M1 and M2 macrophages is thought to either exacerbate inflammatory responses or impair the healing process, contributing to lung injury and the progression of ALI/ARDS (38–41).

The abundance of data from public high-throughput datasets allows robust investigation of ALI/ARDS-associated biomarkers and potential therapeutic targets. Therefore, this study commenced with a bioinformatics analysis from which co-upregulated DEGs were identified across two ALI-related microarray datasets. Functional analysis via GO and KEGG pathways showed that these co-upregulated DEGs were mainly associated with immune and inflammatory responses. The PPI network analysis identified the top 10 hub genes. Consistent with the literature suggesting a pivotal role of immune cell infiltration in ALI/ARDS pathogenesis (42–44), our analyses detected a significant increase in macrophage presence within the ALI cohort compared to controls. Intriguingly, the proportion of M1 macrophages notably exceeded that of M2 macrophages. This imbalance in macrophage polarization provides evidence for the role of these cells in promoting the development of ALI/ARDS. Furthermore, to substantiate these findings, the expression of the identified hub genes was validated at the mRNA level in two external GEO datasets related to macrophages.

*CD274* (PD-L1) is an immune checkpoint molecule that inhibits the host’s immune response against tumor cells (13, 45) and regulates inflammation development (18, 46). PD-1/PD-L1 inhibitors, antibody-based therapies derived from immune checkpoint therapy and clinically approved for various types of cancers, can alleviate T-cell exhaustion and enhance the immune response towards cancer cells (47, 48). PD-L1 blockade has been associated with the development of ARDS-like pneumonitis in cancer patients treated with PD-L1 checkpoint inhibitors. This suggests that *CD274* (PD-L1) may play a role in the etiology of ARDS. However, the exact mechanistic contribution of *CD274* (PD-L1) to the pathogenesis of ALI/ARDS remains to be elucidated, as current studies reveal divergent results. Tu et al. found that human umbilical cord mesenchymal stem cells (HUCMSCs) promote the expression of *CD274* (PD-L1) in macrophages. This results in an increase in PD-1 expression and a reduction in IL-2 and IFN-γ production in T cells. Treatment with HUCMSCs has been shown to attenuate lung injury in mice (49). Xu et al. discovered that soluble PD-L1 (sPD-L1) levels were higher in direct ARDS survivors than in non-survivors. They administered sPD-L1 to mice with ALI and observed a decrease in pro-inflammatory macrophages, which ultimately relieved inflammatory lung injury and improved survival rates (17). In a separate study, lipid nanoparticles carrying lung-targeted sPD-L1 mRNA were administered to ALI mice. The treatment resulted in a notable reduction in leukocyte chemotaxis and protein accumulation in lung tissue, as well as a decrease in pulmonary edema (50). A separate study discovered that individuals with ARDS who underwent prolonged mechanical ventilation or died exhibited significantly lower expression levels of *CD274* gene in AMs (51).

However, the role of PD-L1 in ARDS is still unclear due to conflicting results. ARDS is a significant complication in COVID-19 patients (52, 53). Morrell et al. found that high plasma levels of sPD-L1 were linked to fewer ventilator-free days and higher mortality rates in COVID-19 patients (54). *CD274* (PD-L1) expression increases in severe COVID-19 patients and is associated with cytokine storm and CD8^+^ T cell exhaustion (55). The *CD274* (PD-L1) gene expression is upregulated by SARS-CoV-2, leading to immunosuppression and T-cell exhaustion (56). Anti-PD-L1 therapy has been proposed as a potential treatment to reduce serious complications, such as ARDS, in COVID-19 patients (57). PD-L1 inhibits autophagy and promotes NET release by activating the PI3K/Akt/mTOR pathway, which ultimately exacerbates lung injury (19). Silencing PD-L1 expression on mouse lung endothelial cells attenuates the progression of indirect acute lung injury in mice (33, 58). Therefore, these conflicting findings emphasize the need for further research to clarify the underlying mechanisms and the role of *CD274* (PD-L1) in ALI/ARDS. Additionally, the correlation between *CD274* (PD-L1) and macrophage polarization in ALI/ARDS, as well as the potential diagnostic value of *CD274* (PD-L1), remain understudied.

In this study *CD274* expression was found to be significantly higher in M1 macrophages than in M2 macrophages through bioinformatics analysis. Additionally, ROC curve analysis of an external validation cohort associated with ARDS indicated that *CD274* has potential value in the clinical diagnosis of this disease. GSEA enrichment analysis also revealed an association between *CD274* expression and the JAK-STAT signaling pathway. The bioinformatics results suggest that *CD274* (PD-L1) may be a key gene associated with M1 macrophages in ALI/ARDS. Subsequent *in vivo* and *in vitro* experiments were performed to validate the bioinformatics predictions. The data revealed a significant upregulation of *CD274* (PD-L1) expression in LPS-induced M1 macrophage models, including both RAW264.7 cell lines and bone marrow-derived macrophages (BMDMs), as well as in lung tissues and peripheral blood samples from ALI mouse models. These results corroborate the initial bioinformatics findings, suggesting a pivotal role of *CD274* (PD-L1) in the inflammatory response associated with ALI.

The JAK-STAT3 signaling pathway has been demonstrated to regulate PD-L1 expression in tumor cells (27–29). However, there is limited research on whether this pathway also regulates PD-L1 expression in macrophages during the pathogenesis of inflammatory diseases, such as ALI/ARDS. Our findings showed that levels of STAT3 phosphorylation were significantly elevated in the lung tissues of ALI mice. Additionally, we found that the JAK-STAT3 pathway acted as an upstream signaling pathway, promoting LPS-induced M1 polarization and PD-L1 expression in macrophages.

To determine the role of *CD274* (PD-L1) in the regulation of M1 macrophage activation, we utilized siRNA-mediated gene silencing to knock down *CD274* (PD-L1) expression in BMDMs. Our study revealed that knockdown of *CD274* (PD-L1) significantly promoted LPS-induced M1 polarization and the levels of IL-6 and TNF-α in macrophages. Collectively, these data demonstrate that *CD274* (PD-L1) acts as a negative regulator of M1 polarization and proinflammatory cytokine production in macrophage.

## 5 Conclusion

In conclusion, our study identifies *CD274* (PD-L1) as a critical regulator within M1 macrophages in the pathogenesis of ALI/ARDS through bioinformatics and experimental approaches, which has potential as a novel biomarker for early diagnosis of ALI/ARDS. In addition, we demonstrated that the JAK-STAT3 signaling pathway promotes *CD274* (PD-L1) expression on LPS-induced M1 macrophages. As a negative regulatory molecule, *CD274* (PD-L1) inhibited M1 macrophage function and inflammation, which may ameliorate the course and progression of ALI/ARDS. These findings may provide novel therapeutic targets for ALI/ARDS. Further experiments are needed to extend the current findings and to investigate the regulatory mechanisms of *CD274* (PD-L1) in ALI/ARDS.

## Data availability statement

The original contributions presented in the study are included in the article/**Supplementary Material**. Further inquiries can be directed to the corresponding authors.

## Ethics statement

The animal study was approved by Animal Experimentation Ethics Committee of Soochow University. The study was conducted in accordance with the local legislation and institutional requirements.

## Author contributions

NT: Writing – original draft, Conceptualization, Data curation, Formal analysis, Software, Visualization, Methodology, Investigation. YY: Data curation, Formal analysis, Methodology, Software, Writing – original draft, Validation, Visualization. YX: Formal analysis, Investigation, Methodology, Software, Visualization, Writing – original draft. GY: Data curation, Investigation, Methodology, Validation, Writing – original draft. QW: Investigation, Validation, Visualization, Writing – original draft. CL: Formal analysis, Software, Writing – original draft. ZL: Resources, Supervision, Validation, Writing – review & editing. J-AH: Funding acquisition, Resources, Supervision, Validation, Writing – review & editing.
