# Supplementary material for: CD274 (PD-L1) negatively regulates M1 macrophage polarization in ALI/ARDS
Source: Front Immunol. 2024 Feb 19;15:1344805. doi: 10.3389/fimmu.2024.1344805 (PMC10909908; doi:10.3389/fimmu.2024.1344805)
Supplement: Supplementary file 3 [file Table_1.pdf]

Supplementary Table 1. Detailed information for the GEO datasets in this study.

Dataset	Platform	Species	Experimental group	Control group
GSE2411	GPL339	house mouse	6 (ALI)	6 (healthy controls)
GSE18341	GPL1261	house mouse	8(ALI)	8 (healthy controls)
GSE57614	GPL6480	Human	8 (M1 macrophage)	8 (M2 macrophage)
GSE61298	GPL15207	Human	6 (M1 macrophage)	9 (M2 macrophage)
GSE76293	GPL570	Human	11 (ARDS patients)	11 (healthy volunteers)
