# Supplementary material for: CD274 (PD-L1) negatively regulates M1 macrophage polarization in ALI/ARDS
Source: Front Immunol. 2024 Feb 19;15:1344805. doi: 10.3389/fimmu.2024.1344805 (PMC10909908; doi:10.3389/fimmu.2024.1344805)
Supplement: Supplementary file 4 [file Table_2.pdf]

Supplementary Table 2.

The sequences of genes used in assays of siRNA knockdown

**Target Gene Name**	**siRNA sequences**
CD274-siRNA-1	sence: GAAAUGAUACACAAUUCGATT<br>antisense: UCGAAUUGUGUAUCAUUUCGG
CD274-siRNA-1	sence: CACUUCUGAGCAUGAACUATT<br>antisense: UAGUUCAUGCUCAGAAGUGGC
siRNA negative control	sence: UUCUUCGAACGUGUCACGUTT<br>antisense: ACGUGACACGUUCGGAGAATT
