# Supplementary material for: CD274 (PD-L1) negatively regulates M1 macrophage polarization in ALI/ARDS
Source: Front Immunol. 2024 Feb 19;15:1344805. doi: 10.3389/fimmu.2024.1344805 (PMC10909908; doi:10.3389/fimmu.2024.1344805)
Supplement: Supplementary file 5 [file Table_3.pdf]

Supplementary Table 3. Primer sets used for qRT-PCR analysis.

Gene	Forward Primer (5' to 3')	Reverse Primer (5' to 3')
CD274	TGAGCAAGTGATTCAGTTTGTG	CATTTCCCTTCAAAAGCTGGTC
GAPDH	GGTTGTCTCCTGCGACTTCA	TGGTCCAGGGTTTCTTACTCC
NOS2	ATCTTGGAGCGAGTTGTGGATTGTC	TAGGTGAGGGCTTGGCTGAGTG
IL-6	CTCCCAACAGACCTGTCTATAC	CCATTGCACAACTCTTTTCTCA
IL-1B	CACTACAGGCTCCGAGATGAACAAC	TGTCGTTGCTTGGTTCTCCTTGTAC
CXCL10	CCACGTGTTGAGATCATTGCC	GAGGCTCTCTGCTGTCCATC
CCL2	TTTTTGTCACCAAGCTCAAGAG	TTCTGATCTCATTTGGTTCCGA
TLR2	ATGCTTCGTTGTTCCCTGTGTTG	AGTGGTTGTCGCCTGCTTCC
CXCL1	ACCGAAGTCATAGCCACACTC	CTCCGTTACTTGGGGACACC
CCL3	CCATATGGAGCTGACACCCC	TCAGGAAAATGACACCTGGCT
IFIT3	CTCAGAACCAGTACCTGAAAGT	TTTCTTGTAAAACTGAGCTGCC
IFIT1	AAATTTCCAGAAGGCACTGAAC	TGAACATTCTGACAAACACGTC
CD86	ACGGAGTCAATGAAGATTTCCT	GATTCGGCTTCTTGTGACATAC
CD206	CCTATGAAAATTGGGCTTACGG	CTGACAAATCCAGTTGTTGAGG
ARG1	CATATCTGCCAAAGACATCGTG	GACATCAAAGCTCAGGTGAATC
TGFB1	CCAGATCCTGTCCAAACTAAGG	CTCTTTAGCATAGTAGTCCGCT
