# Supplementary material for: CD274 (PD-L1) negatively regulates M1 macrophage polarization in ALI/ARDS
Source: Front Immunol. 2024 Feb 19;15:1344805. doi: 10.3389/fimmu.2024.1344805 (PMC10909908; doi:10.3389/fimmu.2024.1344805)
Supplement: Supplementary file 6 [file Table_4.pdf]

Supplementary Table 4. The antibodies used in the study.

**Antibody**	**Sources (Catalogue #)**	**Dilution**
PD-L1	abcam (#ab213480) (UK)	WB 1:1000
iNOS	abcam (#ab283655) (UK)	WB 1:1000
GAPDH	Proteintech (#10494-1-AP) (China)	WB 1:10000
STAT3	CST(#9139S)(USA)	WB 1:1000
pStat3 (Tyr705)	CST(#9145S)(USA)	WB 1:1000
JAK1	CST(#3344)(USA)	WB 1:1000
pJAK1(Tyr1034/1035)	CST(#74129)(USA)	WB 1:1000
JAK2	CST(#3230)(USA)	WB 1:1000
pJAK2(Tyr1008)	CST(#8082)(USA)	WB 1:1000
PD-L1	Proteintech (#66248-1-Ig) (China)	IHC 1:5000
CD86	BD Biosciences(#561963)(USA)	FC 1:200
F4/80	BioLegend(#123116)(USA)	FC 1:200
