# Supplementary material for: CD274 (PD-L1) negatively regulates M1 macrophage polarization in ALI/ARDS
Source: Front Immunol. 2024 Feb 19;15:1344805. doi: 10.3389/fimmu.2024.1344805 (PMC10909908; doi:10.3389/fimmu.2024.1344805)
Supplement: Supplementary file 7 [file Presentation_1.pptx]

## Slide 1
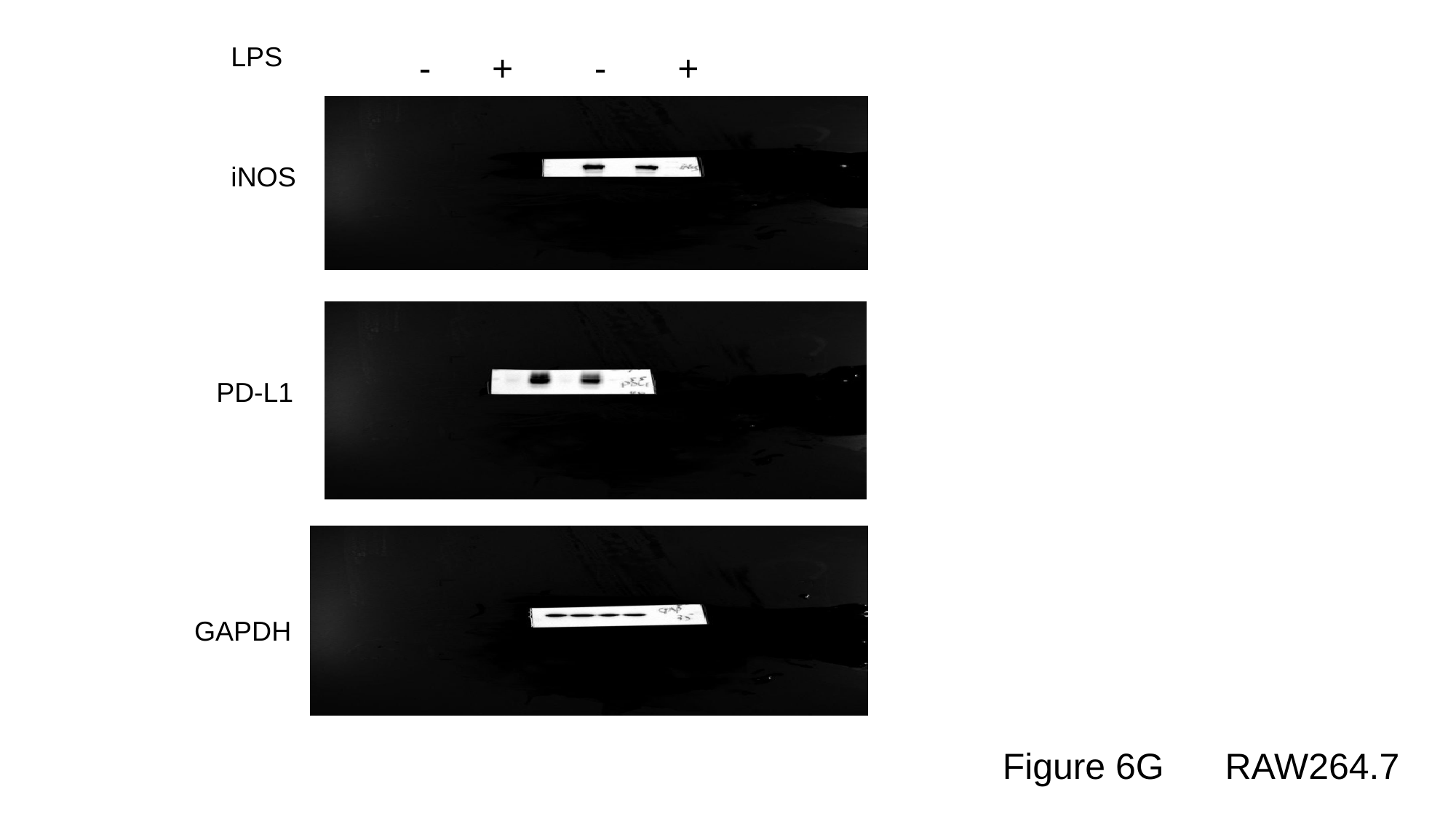

LPS
- + - +
iNOS
PD-L1
GAPDH
Figure 6G RAW264.7

## Slide 2
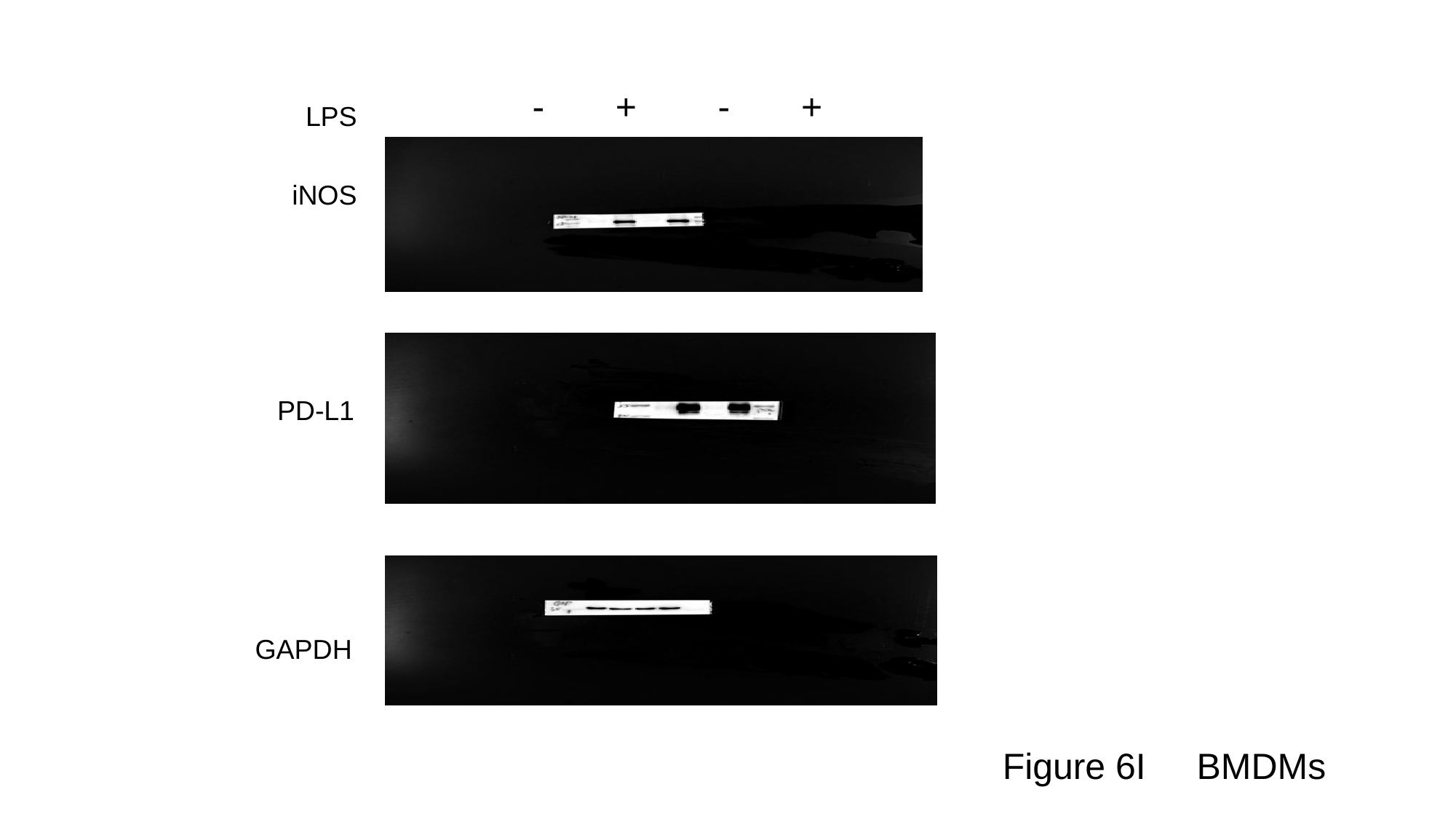

- + - +
LPS
iNOS
PD-L1
GAPDH
Figure 6I BMDMs

## Slide 3
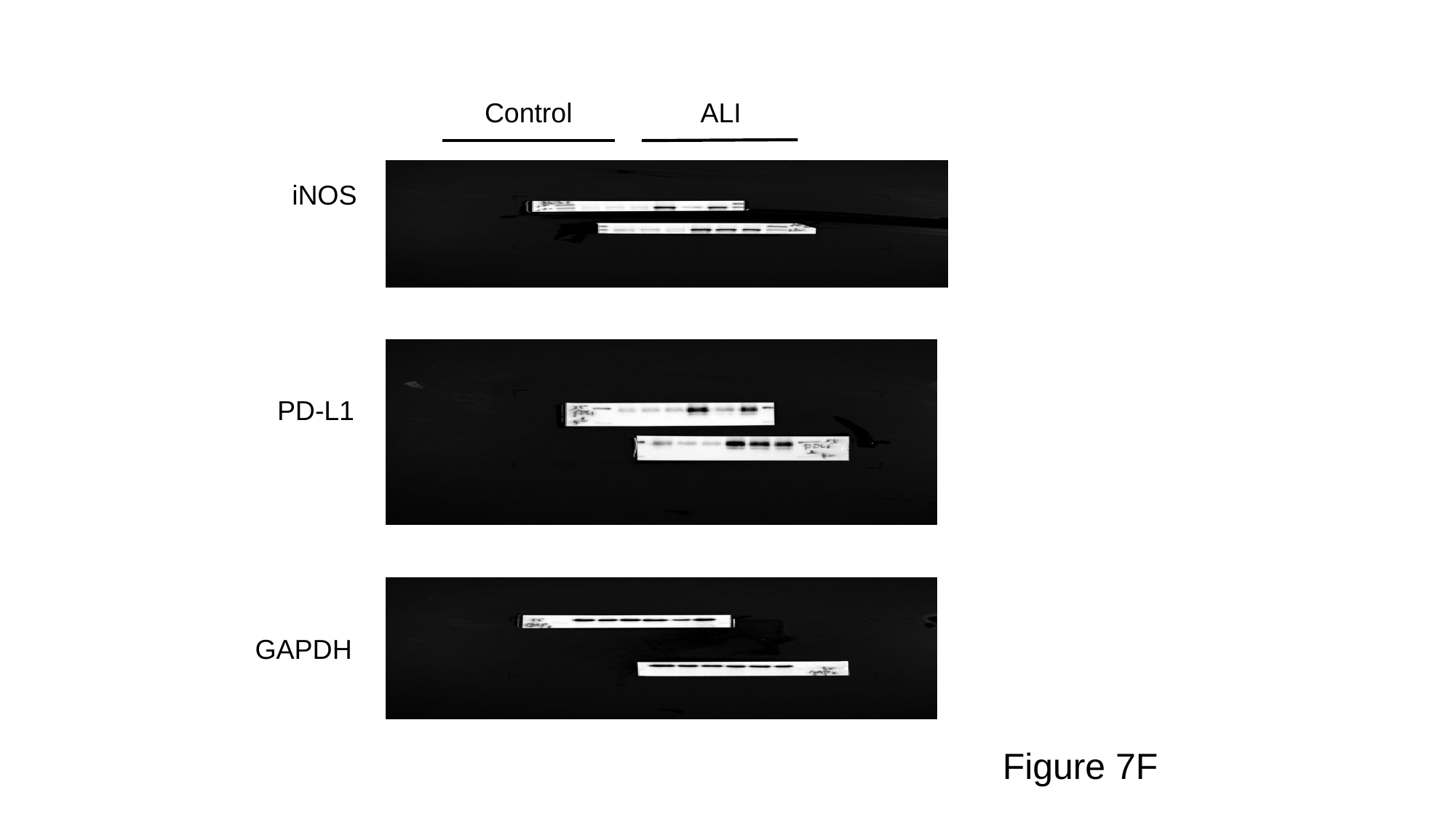

Control
ALI
iNOS
PD-L1
GAPDH
Figure 7F

## Slide 4
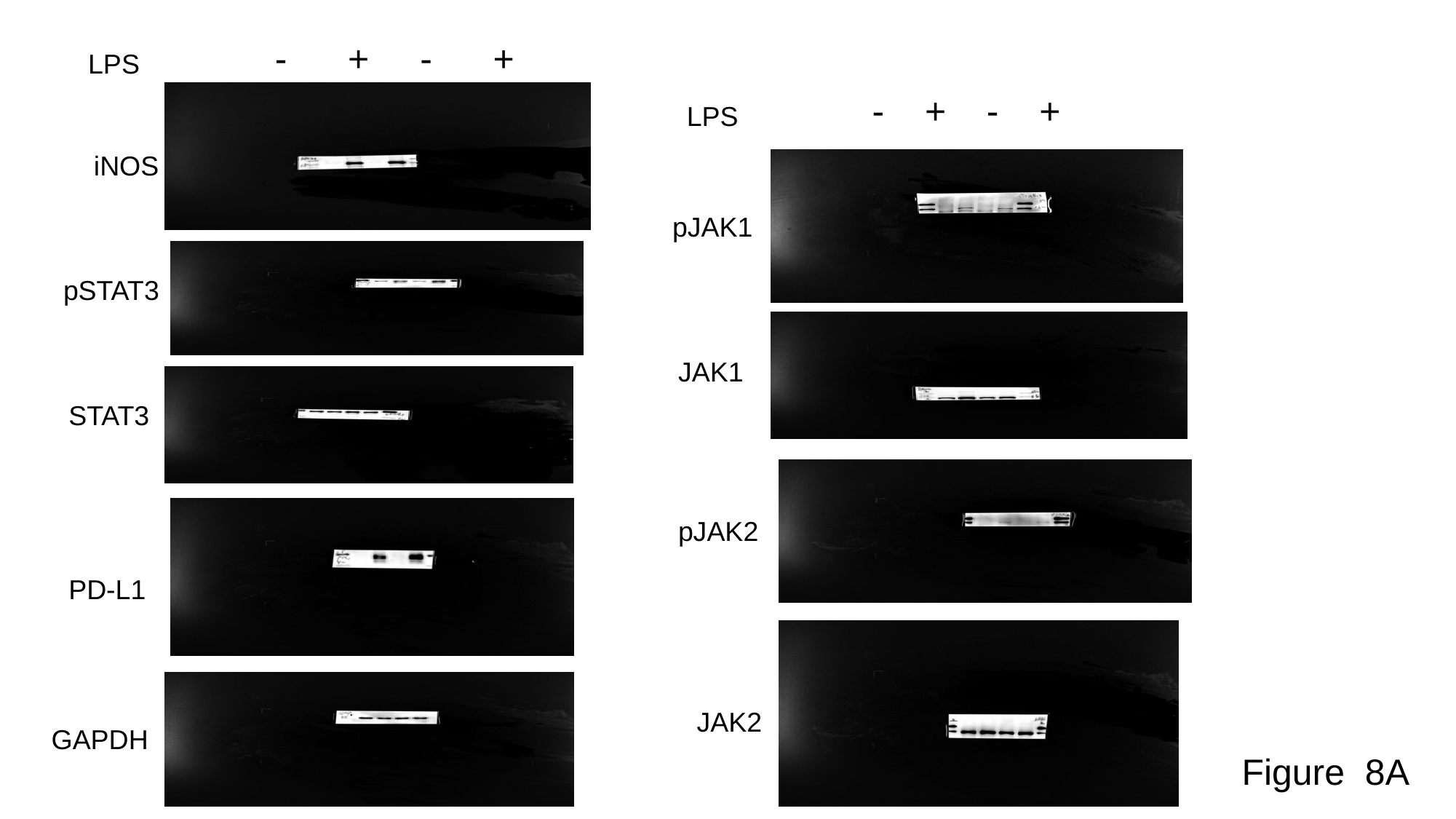

- + - +
LPS
- + - +
LPS
iNOS
pJAK1
pSTAT3
JAK1
STAT3
pJAK2
PD-L1
JAK2
GAPDH
Figure 8A

## Slide 5
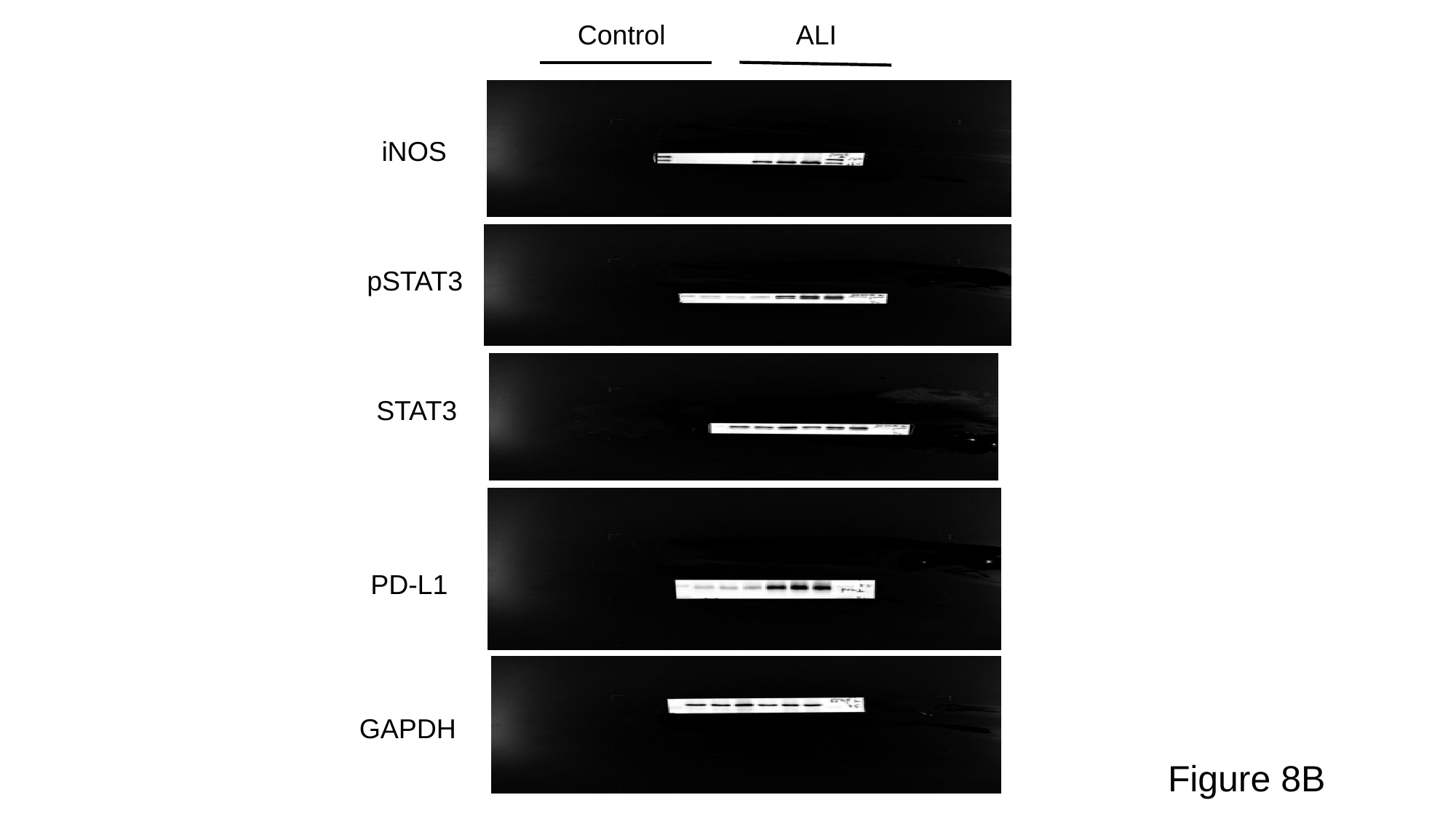

Control
ALI
iNOS
 pSTAT3
 STAT3
PD-L1
GAPDH
Figure 8B

## Slide 6
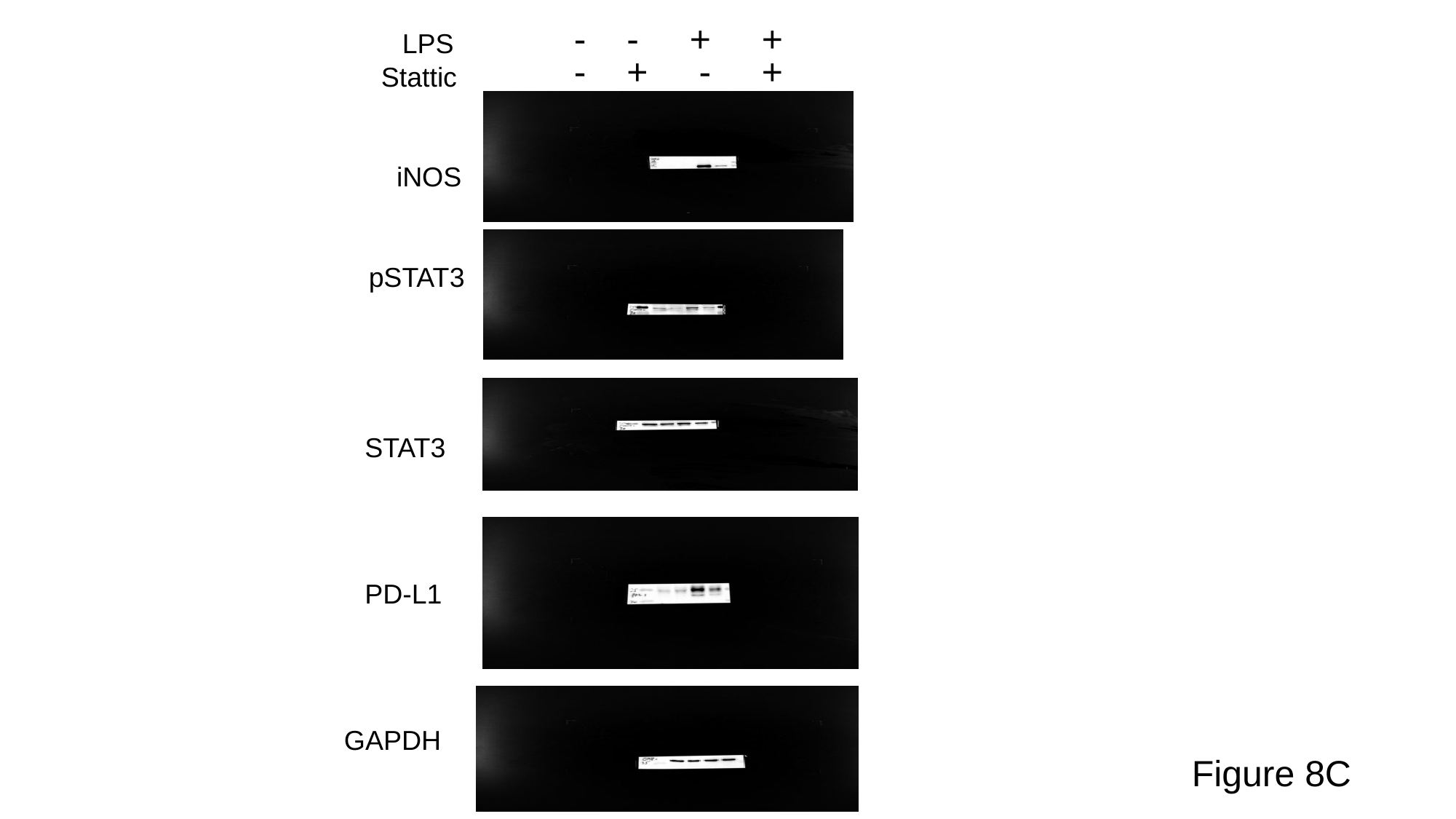

- - + +
LPS
- + - +
Stattic
iNOS
pSTAT3
STAT3
PD-L1
GAPDH
Figure 8C

## Slide 7
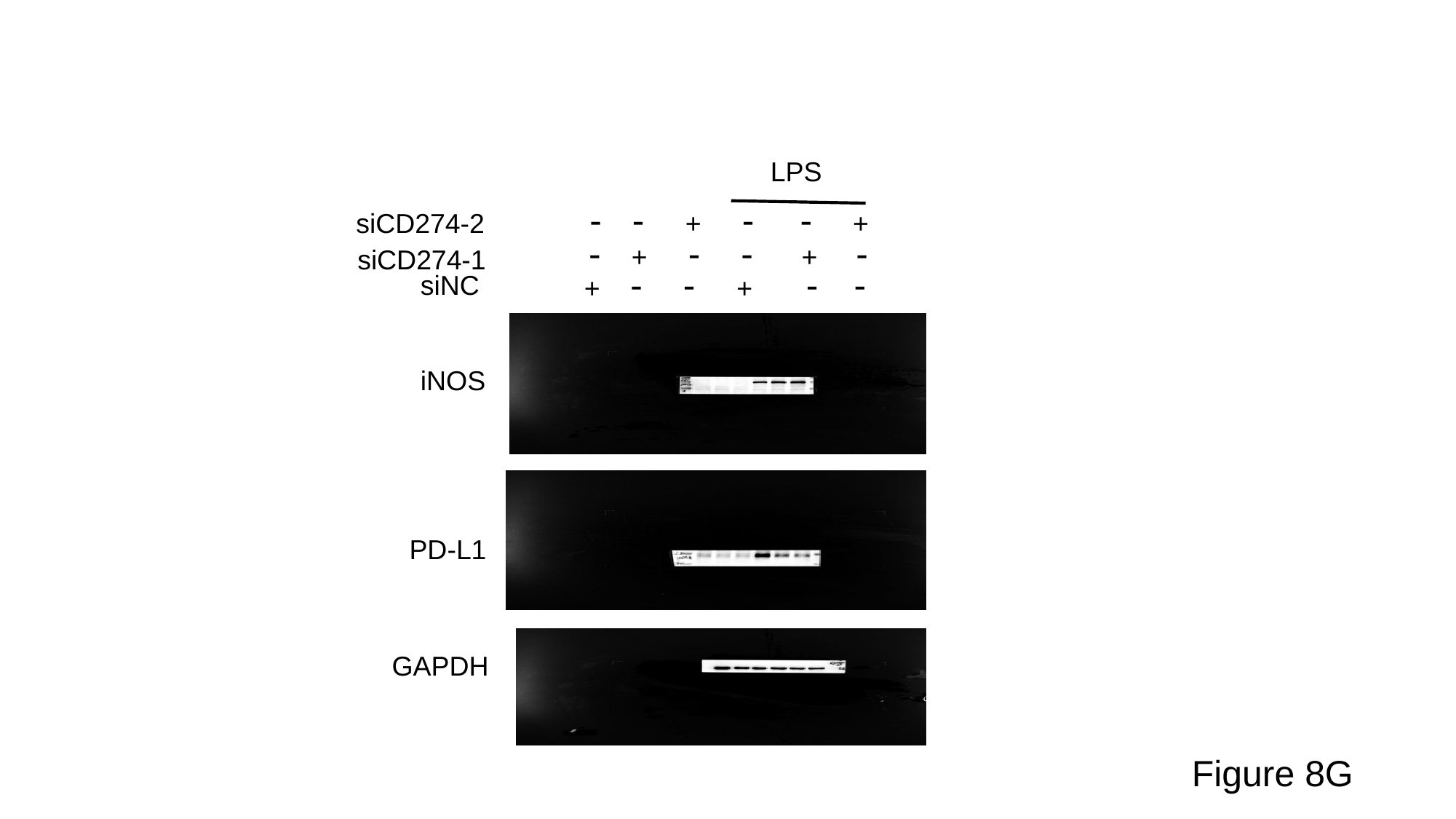

LPS
 - - + - - +
siCD274-2
 - + - - + -
siCD274-1
 + - - + - -
siNC
iNOS
PD-L1
GAPDH
Figure 8G
