# Supplementary material for: CD274 (PD-L1) negatively regulates M1 macrophage polarization in ALI/ARDS
Source: Front Immunol. 2024 Feb 19;15:1344805. doi: 10.3389/fimmu.2024.1344805 (PMC10909908; doi:10.3389/fimmu.2024.1344805)
Supplement: Supplementary file 8 [file DataSheet_1.docx]

Supplementary Material

# Supplementary Figures and Tables

## Supplementary Figures

**Supplementary Figure 1.** Validating hub genes expression in LPS-induced M1 macrophages (RAW264.7) by qPCR.

**Supplementary Figure 2.** Identification macrophages by detecting F4/80 using FC

## Supplementary Tables

**Supplementary Table 1.** Detailed information for the GEO datasets in this study.

## Supplementary Table 2. The sequences of genes used in assays of siRNA knockdown.

## Supplementary Table 3. Primer sets used for qRT-PCR analysis.

## Supplementary Table 4. The antibodies used in the study.

# The original images of Western blot
